# Exploring the general practitioners’ point of view about clinical scores: a qualitative study

**DOI:** 10.1186/s41512-023-00149-x

**Published:** 2023-06-13

**Authors:** Maxime Pautrat, Remy Palluau, Loic Druilhe, Jean Pierre Lebeau

**Affiliations:** 1 Faculty of Medicine, University of Tours, EA7505 Education Ethique Santé, 10 boulevard Tonnellé, 37000 Tours, France; 2 Department of General Practice, Tours Regional University Hospital, Tours, France

**Keywords:** Decision aids, Psychometrics, Primary health care, Qualitative research

## Abstract

**Background:**

Clinical scores help physicians to make clinical decisions, and some are recommended by health authorities for primary care use. As an increasing number of scores are becoming available, there is a need to understand general practitioner expectations for their use in primary care. The aim of this study was to explore general practitioner opinions about using scores in general practice.

**Method:**

This qualitative study, with a grounded theory approach, used focus groups with general practitioners recruited from their own surgeries to obtain verbatim. Two investigators performed verbatim analysis to ensure data triangulation. The verbatim was double-blind labeled for inductive categorization to conceptualize score use in general practice.

**Results:**

Five focus groups were planned, 21 general practitioners from central France participated. Participants appreciated scores for their clinical efficacy but felt that they were difficult to use in primary care. Their opinions revolved around validity, acceptability, and feasibility. Participants have little regard for score validity, they felt many scores are difficult to accept and do not capture contextual and human elements. Participants also felt that scores are unfeasible for primary care use. There are too many, they are hard to find, and either too short or too long. They also felt that scores were complex to administer and took up time for both patient and physician. Many participants felt learned societies should choose appropriate scores.

**Discussion:**

This study conceptualizes general practitioner opinions about score use in primary care. The participants weighed up score effectiveness with efficiency. For some participants, scores helped make decisions faster, others expressed being disappointed with the lack of patient-centeredness and limited bio-psycho-social approach.

**Supplementary Information:**

The online version contains supplementary material available at 10.1186/s41512-023-00149-x.

## Background

Evidence-based medicine (EBM) integrates individual clinical expertise, patient values, and best available evidence from systematic research to make health care decisions [1]. In providing EBM, the general practitioner (GP) is “primarily responsible for providing comprehensive and continuing care” integrating “physical, psychological, social, cultural, and existential factors” [2]. In 2014, it was found that patients consult GPs for an average of 2.6 reasons per consultation [3]. To answer these numerous demands, GPs have to make efficient use of the resources the health system offers to make decisive decisions while in indecisive situations [4].

Clinical scores are tools designed to assist EBM decision-making processes. A score combines relevant clinical or paraclinical items, in a structured manner. The numerical score result reflects the probability of a diagnosis, a prognosis, a symptom, or a disease intensity [5, 6]. A score with strong internal validity, based on the psychometric attributes, reflects its ability to predict an exact result [7]. A score with a strong external validity reflects the replicability and transposability of score criteria [8]. Theoretically, these scores should enable decision-making processes to be standardized, replicable, and explicit, and in so doing, harmonize clinical practice [5].

The number of available scores has increased dramatically in recent years. Specific, online access to scores has increased from 13,500 in 2010 and 25,000 in 2019 [9]. In primary care, clinical scores have been developed for use in geriatrics [10], psychiatry [11, 12], cardiology [13] or surgery [14] and some current guidelines support their use [15], despite many not being validated in primary care [16]. Although, GPs are increasingly using scores, one study reported an increase from 35.2% in 2003 to 75% in 2010 [17, 18]; yet, many scores may be unfeasible for use in general practice and GPs are unaware of the variety of scores available. One report found GPs are aware of at least six scores but only use four [19]. This may be due to lack of knowledge, training or time, doubts towards their usefulness, access or remuneration issues, or a negative impact on the patient-practitioner relationship and poor patient acceptability [18, 20].

Despite this increased interest for using scores in primary care, little research about score feasibility in primary care is available. This is important because designing scores for primary care use should consider the specificities of the primary care setting and GP expectations for using scores in their everyday practice.

The aim of this study was to explore the GPs’ point of view regarding their use of clinical scores in their daily practice.

## Method

### Study design

This qualitative study, with a grounded theory approach explored GP opinions about using clinical scores, based on previous experience. This method was chosen because it ensures that participants verbalize and present their opinions freely [21, 22]. Using a grounded theory approach, enabled investigators to build a model of the GP expectations for suitable scores.

### Participants

GPs practicing in the Loire Region, France, were recruited according to predefined criteria elicited from a preliminary literature review [23] including age, gender, practice type (alone or within a group), setting (urban, suburban, or rural), number of appointments per hour, medical conference participation or continuing medical education, and complementary activities. The investigators called potential GPs to organize a focus group and then recruited additional GPs in the surrounding area using a snowball approach. Recruitment was completed throughout the research project using purposive theoretical sampling, as the emerging theory evolved. The sample size was closed after a focus group with few news ideas and a final focus group of confirmation which did not add more data, according to the sufficiency data principle [24].

### Data collection

Focus groups were planned to collect the data, which were conducted in participating GP surgeries. Focus groups were chosen to enhance the dynamic exchange between GPs about their practice habits [24]. MP, a male GP and specialist in qualitative research, and RP, a male medical student who had received qualitative research training, conducted all the focus groups. There was no relationship between participants and the researchers prior to study commencement. Prior to the focus groups, the participants received information about the research and the researchers and provided written informed consent. Furthermore, the researchers explained their interest in the research topic at the start of the focus group. A semi-structured interview framework was developed to direct focus group discussions with open-ended questions that encouraged discussion (Additional file 1). This initial framework was progressively enriched with significant elements elicited from previous focus groups, according to an inductive analysis process. Sometimes, discussions were enhanced with examples of tests recommended for use in primary care, with a variety of multi-topic questionnaires, and shorter (CRB65) and longer tests (FINE) [25–27]. Field notes were made during the focus groups. Each focus group was audio recorded, fully transcribed, and anonymized. Participants could request to see the transcripts. Only participants and the two interviewing researchers were present during the focus groups.

### Data analysis

At the end of each focus group the verbatim was blinded. Two experienced investigators separately performed the analysis to ensure analyst triangulation using a coding tree. The investigators labelled the verbatim in a double-blind fashion and individual labels were then combined into a single label through discussion and arbitration with a third investigator whenever needed. Labelled items would then be categorized in an inductive way using the same double-blind and arbitration process. QSR NVivo11® software was used for data collection and analysis. Participants received RP’s thesis and were able to provide feedback on the results.

## Results

**Fig. 1:**
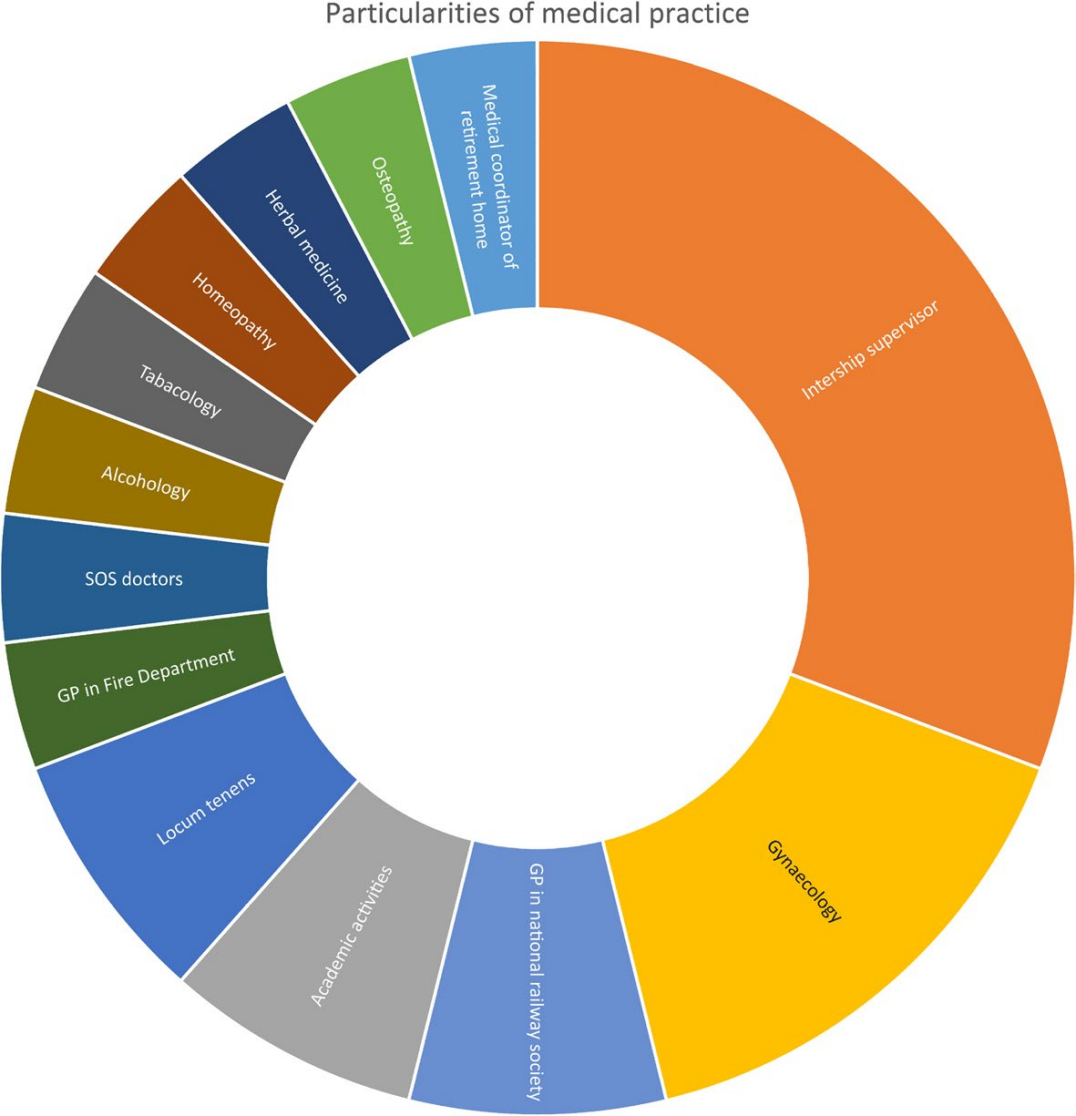
Particularities of medical practice

Between August 2017 and June 2018, five focus groups were planned but data saturation was achieved after the fourth focus group. In all, 21 GPs participated, no GPs refused to participate or dropped out. The focus groups lasted for an average of 60 min. The focus groups and participant characteristics are described in Table 1 and Fig. 1. We found that opinions expressed regarding clinical scores revolved around the three major safety and quality characteristics of an intervention; validity, acceptability, and feasibility [28].

**Table 1:** Participant and focus group characteristics

	Focus 1	Focus 2	Focus 3	Focus 4	Focus 5	Total
Duration	00:58:11	00:49:13	00:56:16	01:00:03	01:18:41	05:01:24
Number of participants	4	5	4	4	4	21
Men/women	2/2	3/2	3/1	2/2	2/2	12/9
Average age [min–max]	42 [30–62]	38 [27–66]	44 [33–58]	44.5 [34–52]	41.5 [29–62]	41.8 [27–66]
Number of consultations per hour	3,5	4	3,5	3,37	3,25	3,55
Participants in conferences	1	3	2	0	1	7
Activity						
Established	4	4	4	4	3	19
Locum	0	1	0	0	1	2
Practice type						
Group	4	3	4	4	1	16
Single	0	1	0	0	2	3
Location						
Rural	4	4	0	0	0	8
Peri-urban	0	0	0	4	1	5
Urban	0	0	4	0	2	6

### GPs have little regard for score validity

#### Score popularity prevails over scientific validity

GPs reported favoring scores more for their popularity in the medical community than for their validity: “I have been using it since I was a medical student and I never really thought twice about it”. Some GPs assumed that the fact that they are using a score gives credit to their practice: “I found that it puts things in order on paper”.

#### Scores are more distrusted than trusted

GPs criticized some scores for not being designed for outpatient practice: “it is unusable; it’s more of an emergency department thing”. Also, they doubted the reliability of their own interpretation of score results: “I think that we can underestimate or overestimate score item values while actually using it”. The GPs also questioned the validity of a given score for subjective symptoms, such as in depression: “The HAMILTON scale is up to the interviewer’s estimation and not the patient’s. You’re not going to ask the patient ‘have you ever, rarely, or never’”. “It’s up to you to decide”. “I found it difficult to evaluate this”. It is hard to limit a symptom to a number without losing all its nuances: “The score item doesn’t match with the information you have in the first place. Then you end up mixing things that have nothing to do with one another in the same clinical score”. GPs also highlighted the lack of background information captured with a score. GPs know their patients’ personal background, which is difficult to quantify; “I especially need the context”. GPs reported “losing pieces of information” with a number alone. They never have “a blind trust in clinical scores”, “if elements are lacking, I’m going to consider it as invalid and then I cannot rely on it to make a decision”.

### GPs report many scores are difficult to accept

#### Clinical scores are robotic, but general practice is a human science

For some practitioners, merely the thought of using a clinical score was unacceptable: *“*The very word “score” tends to get on my nerves”. The idea of replacing a human relationship with pre-defined interactions by an algorithm was unacceptable for them: “This is the antithesis of general practice”, “medicine isn’t this; it’s a human science. It’s about our free will, our thoughts, our sensitivity and our knowledge, or else we just turn into computers”.

GPs were disappointed with the rigidity of score questions: “in all those questionnaires, the answers are a bit artificial, because our own attitude is artificial. It’s not the practitioner speaking anymore but a person reading a pre-prepared text with words that may not have used”. The GPs felt uncomfortable with pre-defined questions that replace their own routine: “it totally rips out the dialogue, patients feel like they are talking to a robot”, “the patient-practitioner relationship turns into mathematics”. Lastly, they were shocked by the *“robotic”* sounding names for the clinical scores: “The fact that someone decided to call a score for depressed patients “PHQ9”, I have no words”.

#### Patients expect a good listener, scores expect a checked box

“A depressed person needs to be listened to, not asked questions”. When listening to a patient, manner appears to be crucial for GPs: “you look at the person, you don’t check boxes”. They dreaded going from question to question without adapting to the patient answers: “we are asking questions, yes, but in an order that goes well with the consultation flow, and not a thing recited monotonously, this question then this one, and this one”, “you adapt your speech to the patient!”. GPs didn’t want “to be a lie detector” or be perceived as such. “If you have to ask trick questions… Well, I don’t want to do that”.

### The expense of administering a score in primary care is an issue

#### Clinical scores take time, and time is money

GPs were uncomfortable charging specifically for performing a clinical score: “I didn’t even know that we could do that”, “I just don’t know how to bill for it” and “to search for the billing codes, this annoys me”. The participants debated charging for time spent performing a score: “even though I did it, I’m not going to ask for more because of that” and “If you are at a point where you tell yourself: well I spent half an hour so if I [also] bill that score I’m going to earn more”, on the other hand “it is still a small reward when you [go beyond the level of duty] for 45 min…” and “this is our bread and butter! I don’t do it to be famous!” Apart from this notion of charging for time spent, some participants reported performing a score for remuneration: “The value of the test is the money!” and “honestly this is more for the money than for the patients since it is questions that we already ask ourselves”. However, GPs feared that remuneration would generate too many inspections: “one is bound to be questioned by the social security office. It’s better to keep track”.

### Scores are unfeasible for use in primary care

#### GPs can no longer see the wood for the trees

In general, the participating GPs could only recall a few clinical scores: “I know five that I can quote”. They also argued that the names given to clinical scores makes them hard to remember and claimed that: “it would be easier to find it back on the internet if they didn’t have those silly names”. The GPs were also exasperated things were changing too fast: “it keeps changing!”, “they are proliferating” and “I already feel lost when it comes to clinical scores because there is always a new one”. Some were expecting the learned society to sort it out: “I look at it a little bit closer now that there are the recommendations”.

#### A clinical score is like a GPS: it’s when you need it that you forget to bring it

GPs said they forgot about scores that they don’t use regularly: “if we don’t use them, then we forget about them” and “there were 4 or 5 that I managed to remember and that I used frequently thus they remained in my practice”. And yet it is in rarer pathologies that the help of a tool was more needed: “With rarer pathologies, clinical scores can, maybe, be more interesting. But I can’t quote any score for a rare pathology”.

#### When scores are short, they are inadequate but when they are long, they are useless!

GPs preferred using short scores: “it has to be 3 or 4 questions long”, “yes or no questions, closed questions…”, “rapidly interpretative” with simple questions: “we are doctors, so it is understandable [for us], but there are some sentences that are incomprehensible [for the patient]”. However, they admitted that shorter scores [CRB65] don’t capture contextual elements; “there is also the family background”. Yet more complete scores seemed impractical to them: “adding the context is going to give you another which is going to be more complete but unusable” and “the ideal score is one that is easy to use” and not those “where we don’t have the results in general practice” such as “blood gases”.

#### Scores can be more efficient to administer if someone else fills them out

Clinical scores are not seen as a priority: “I’d rather spend more time doing other things”. For those who use scores, they organized themselves specifically to have time to complete them: “I make them come just for that”, “it’s an appointment, I book them at a specific time”.

Some practitioners suggested that patients complete a self-administered questionnaire to have the patient face his own responsibility: “this way they can look in the mirror, and that can make them realize a few things”. They found that this saved time: “it also allows me to defer a consultation, not to overload one that is already [long]”. However, they debated which was the best way to make self-administered questionnaires acceptable: “you either give it to everyone in the waiting room as you used to do with alcohol, or you don’t, otherwise it’s a little bit like appearance discrimination”. Targeted screenings “such as the blood pressure self-measurements” were also suggested. Others considered developing e-health as a solution: “On [an app] you would have a form, a kind of questionnaire, for new patients to fill out”. Yet the doctors wondered how the patient would react: “mostly they are not going to get it”, being afraid that patients “end up alone [faced with] all those questions”. They also felt that patient implication in this type of score which is confirmed by a participant who had implemented self-questionnaires: “I have a less than 1% feedback”. Some practitioners delegated scores to other health professionals: “I prefer to delegate to someone else”, “I save time, I don’t do it [myself]”, “I send them to the specialized nurse”, “you give them to the social worker”.

### GPs believe scores are relevant in some situations

#### Clinical scores are to doctors what Morse code is to sailors

Clinical scores were considered by some practitioners as a communication tool between professionals: “it’s difficult to relay written information”, the point was “to have a standardized measure, numbered, something that can be understood at the other end of the line”. GPs used clinical scores as a simple tool to give information to the patient in a split decision situation: “he didn’t make a decision, it was the patient that had to make it, so we showed him his score results and there you go”. Other practitioners saw scores as a way to tackle delicate issues: “the aim is to open the discussion on questions that we might not ask”, including through self-administered questionnaires; “they fill them out, then if they end up with the result: “big problems” maybe it’s going to push them towards their GP one day”.

#### Young doctors trust clinical scores while experienced doctors trust clinical judgement

GPs criticized clinical scores for not considering the context, which younger practitioners found stressful: “you’re already very organ-centered [when coming out of medical school], and yet you put scores on top of this”. Some GPs tended to use scores that they recently discovered: “you have a new grid to fill out, you’re happy, you add this in your professional credentials”. For others, scores can fall into oblivion: “I did none this year, I forgot about it”, “and then in practice, time flies, you tend to smooth your [practice] out and reproduce the old patterns”. Scores were progressively included in consultations without being formally implemented: “you start to know the questions so well that you’re using them during your consultation without putting any result number at the end”. Practitioners relied on their own experience rather than scores alone: “perception, remains the best thing for those cases”, “in the decision-making process we rely on our clinical judgement, and not a score, which is not going to tell us what to do” and “we have a certain semiology in our head, a much-vaunted «sense of alarm», clinical experience, and we don’t need to check boxes or to calculate”. This feeling seemed to grow over time: “I asked myself, in the end, what do we want from the tests? What is their use? And then I think I realized, with time, that I didn’t really need them because they weren’t of much use”.

#### No treatment, no test

GPs used scores that “changed something in the end”: “If we already know what needs to be done then we don’t do the test” and “if we take a memory loss problem, either scoring or not, the progression will be what it is, and the scales won’t change a thing”. GPs expected a score to add value, “bring something more to the table, an added value in comparison with daily practice”. The GPs expected a score to improve decision-making “in COPD or tonsillitis, scores help me decide whether or not to prescribe an antibiotic”; improve their care “It needs to help us with the diagnosis, or administer a treatment”, or provide patient information “It needs to provide us with information to convince the patient!”.

#### Clinical scores give way to catastrophism

Faced with doubt, practitioners did not feel clinical scores were reassuring: “I don’t believe they would reassure me that much”, “the test will still leave me alone with my uncertainty”, and “even if there was a reassuring test, I would still call saying that I’ve done the test and it looks reassuring, but I still don’t feel good about it”. Instead, an alarming score result led to more screening tests: “if the result number is really high, I tell myself that I’m missing something”. In the end, for the GP, “from a legal point of view it can protect us” but for the patient, “you’d just say that you’re sorry he is dead but he got 1 on the test score, it wouldn’t change a thing”.

#### Clinical scores are to monitoring what a dermascope is to a melanoma

Participating GPs used scores to monitor their patients: “I sometimes found it useful for monitoring because people would tell you that things are not getting better, but then when you do the test a second time you figure out that there has been an improvement”. They stressed reproducibility: “the scale needs to be reproducible” and “allow you to measure progression”, in terms of test-retests reliability. The value is increased when several medical professionals are monitoring the same patient: “it is not always the same person that sees the patient in the hospital”.

#### No pathway specialization without research, no research without clinical scores

GPs thought that research in primary care needed clinical scores: “for someone who’s going to study something, then these tests have significance for evaluations”. The tool brought forth necessary numbered data: “it can be biostatistics. It’s good because it can give you pointers on population health conditions and their practices”. This positive side was well perceived for general practice as a discipline, but less for their own practice: “it’s true that if we were to perform research in general medicine, clinical scores won’t help us with clinical judgement but may boost some [health] indicators”. Nevertheless, GPs did not feel involved in this process: “If it’s for research purposes then we are not directly in it”.

## Discussion

This study explored GPs’ opinion about their use of scores in their current practice. GPs expressed often seeing little value in using clinical scores. They tended to value relevance and feasibility over scientific validity but felt that scores didn’t take into account clinical circumstances and patient preferences, which are essential elements of EBM.

**Fig. 2:**
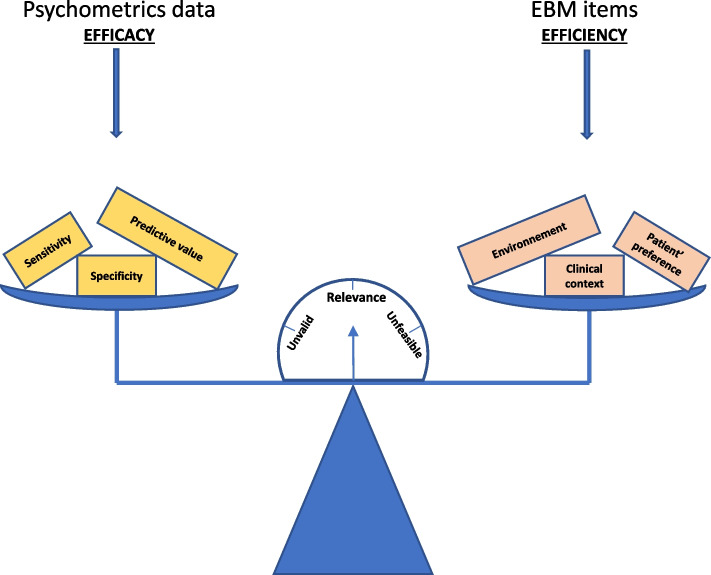
Conceptualization of clinical score properties for decision-making in primary care settings

The findings in this study challenge using scores to provide EBM in general practice. Although GPs appreciated that scores have psychometric efficacy for use as good communication tools, or are a reference point for current scientific data, they questioned their efficiency in primary care. GPs clearly expressed concern about excluding patient preferences, using a robotic-like approach or box checking to obtain background context. A recent review suggested that a score could have some positive effect on process outcomes but their results may be context specific [29]. These elements are nonetheless necessary to the Engel’s model of bio-psycho-social approach [30]. The practitioner must therefore deliberate between the score psychometric efficacy and efficiency, before deciding to use it. The findings from this study conceptualize the efficacy-efficiency balance to guide which clinical scores are suitable for use in general practice (Fig. 2).

### Feasibility and relevance reflect the efficiency-efficacy balance

Efficient scores are those that are feasible and easily administered and influence patient management, which may encourage GPs to adopt scores in general practice. Currently, two scores meet these needs: the Ottawa score which confirms the need for an X-ray in only three questions for a twisted ankle, and the BITS test which points to suicidal risks for a teenager with four questions [31, 32]. Both scores have been developed by primary care practitioners which enhance their relevance. In contrast, the CHA_2_DS_2_VASC score to evaluate the need for an anti-coagulant treatment in atrial fibrillation, is also a short score [33]. Yet, the CHA2DS2VASC is seen as being “for hospital specialists” and inefficient for primary care: “in the end, the patient will still always end up at the cardiologist”. This perception was reiterated in a recent systematic review confirming that relatively few potentially relevant tools for primary care have undergone impact analysis, and their implementation has been restricted to a limited number of clinical domains, mainly musculoskeletal, cardiovascular and respiratory [34]. Also, even tools designed for primary care have not undergone impact analysis, making it difficult to obtain a significant increase in sensitivity or specificity in treatment decision-making between score-assisted and non-assisted clinical skills.

### Validity, affordability, and acceptability

GPs were less concerned with the validity of the score than with the appropriateness of the test in their practice. Some GPs reworded validated tests to administer the test faster or reduce perceived patient discomfort. However, doing this has been shown to decrease their quality [35, 36]. Furthermore, participating GPs wanted to maintain human relationships and not rely on a standardized test, which has also been supported by the literature [17]. GPs are encouraged to use the mini mental state examination (MMS) for early diagnosis of dementia even though the inter-reliability is limited. GPs can correctly identify individuals with cognitive impairment, even if they scored their patients lower than the Alzheimer specialists scored.

Despite score use depending upon GP remuneration, it is considered encouraging that scores are used at a European Level [37, 38]. In France, the MMS or the Hamilton scale are among the top three most commonly used scores, and GPs can charge up to three times the standard price.

### Professional experience reduces the interest given to clinical scores

We found mixed results concerning the value of scores with increased experience. We found more experienced GPs seemed less inclined to use scores, especially in situations they believe they have mastered, relying more on their gut feeling [39]. These results corroborate other work about GP expectations for scores to improve the decision-making process (diagnostic, complementary explorations, therapeutics) [40]. Other practitioners feel scores are valuable in unusual situations in which they are uncomfortable. However, GPs still admit that for those rare occasions they are often unaware of an appropriate score.

Participants thought that clinical scores could be helpful for the decision-making process among practitioners lacking experience such as newly qualified doctors or medical students: “it’s interesting in training, it’s true that it can help you ask yourself the right questions, remember the priorities”.

### What is the role of clinical scores in general practice?

The GPs mentioned using scores to justify making medical decisions, particularly when healthcare professionals disagreed about the state of a patient in their care. They argued that although the psychometric features may be imperfect, were they better than the GP’s? They expect “irrefutable proof that scores improve the quality of healthcare”. Yet, a 2002 study about the systematic use of Numeric Pain Rating Scale in primary care concluded that scales did not affect chronic pain relief [41]. GPs were afraid of making mistakes by just relying on scores. We also found opinions similar to a recent National commission for data protection report about artificial intelligence that developing “algorithmic systems eroded individual vigilance” or also that “developing these technologies can affect human identity and dignity, its liberty and its responsibility” [42].

### Study limitations

Although this study is limited by the inherent qualitative design, it is strengthened with a grounded theory approach and focus groups. This option was chosen instead of individual interviews to stimulate the emergence of a wide range of opinions from the GP community and pool their current practices. Despite this, social desirability bias may have occurred if some practitioners did not report their practice truthfully through fear of peer judgment. To minimalize this social desirability bias, the focus groups were chosen from nearby surgeries, among practitioners who already knew each other. By knowing each other well, the GPs also know each other’s practice and so we expected they would have been less likely to be untruthful. Furthermore, in our attempt to limit social desirability, we may have induced the possibility of “group think”. However, the sample was heterogenous and varied in terms of setting and age. We believe that this was sufficient to limit this effect. This resulted in free and convivial discussion. The level of comfort was so much so that unexpected opinions arose, indicating that all participants felt sufficiently at ease to speak freely.

One example, we recorded was a conversation about a conspiracy theory centered on unauthorized use of health data gathered through clinical scores to benefit big pharmaceutical companies, government, or assurance companies: “it’s information, big data, it’s a society issue, not a medical one. The sprawling society is sucking up data from everywhere…from our smartphones. You have to be really careful about this”. These unexpected results echo current societal preoccupations which led to a National commission for data protection report in December 2017 [42]. Two main principles emerged: the first stipulating that the algorithms needed to serve the using doctors, and the other stating the importance of ensuring algorithms do not dictate clinical decisions.

We chose not to restrict the list of scores to obtain an overall point of view from the GPs, independent of their individual situation and score experience. This choice to analyze focus group data using the grounded theory method gave us the opportunity to conceptualize general score use in primary care. In contrast, other studies described GP use of a restricted number of scores, which tightened the exploratory fields [17, 21]. These studies used questionnaires or individual interviews with thematic analyses, or literature review.

The COREQ criteria [43] for grounded theorizing research were respected at each stage.

### Perspectives

#### The imperious necessity to sort out clinical scores

Participants told us that they rarely checked the validity level for scores they used, instead reproduced current practices within their professional network. This means they rely on learned societies to select and recommend relevant scores. Learned societies selecting scores has been previously raised and is influencing the future of score use in general practice. Having too many scores makes it difficult to find the most appropriate score for the situation which leads GPs to give up using them [44]. Clinical scores can also be selected from primary care research, such as an inventory conduced in 2015 for cardio-vascular pathologies which found 10 were validated in general medicine among the 26 clinical scores inventoried [7].

#### Can e-health rescue efficiency?

To facilitate clinical score access, participating GPs suggested some solutions. Electronic tools such as medical software, smartphone apps or websites could be developed. But electronic scores should be well organized and easy to access. Our results also echo previous research suggesting practitioners need to be trained to use scores in everyday practice [45]. Other propositions include self-administered questionnaires that patients complete when making online appointments. These questionnaires could then provide GPs with targeted health data before the appointment with would give them time to discuss health matters on a deeper level. A study suggested also using the waiting room to collect data through self-administered questionnaires [46].

## Conclusions

The efficacy of clinical scores still needs to be optimized for primary care use, but their efficiency will always be an issue even though new technologies may solve some issues. Thus, using clinical scores in current primary care remains a challenge as it relies on an efficiency-efficacy scale.

## Supplementary Information

**Additional file 1.**

## Data Availability

The full dataset is available from the corresponding author.

## Abbreviations

EBM: Evidence-based medicine
GP: General practitioner
MMS: Mini mental state examination
