# Supplementary material for: Exploring the general practitioners’ point of view about clinical scores: a qualitative study
Source: Diagn Progn Res. 2023 Jun 13;7:12. doi: 10.1186/s41512-023-00149-x (PMC10262349; doi:10.1186/s41512-023-00149-x)
Supplement: Supplementary file 1 — Additional file 1. [file 41512_2023_149_MOESM1_ESM.docx]

**Initial outline of Focus Group**

**Presentation:**

Subject and method

1. **Tell me the last time you used a clinical score ?**

Revival:

- Why this one?
- Do you use others for this same situation?
- In what context?
- Do you do it systematically?
- Do you use other clinical prediction rules?
- How did you know this score?

Items to discuss:

- Number of scores used
- Frequency of use

1. **What benefits do you get from using clinical prediction rules?**

Items to discuss and associated revival:

- Follow-up:
- Diagnosis:
- Screening / prevention: Begin a discussion?
- Guide patient: justify a therapeutic management?
- Standardizes the clinical examination?
- Do you use scores in case of follow-up for subjective symptoms, like the pain VAS?
- Do you use scores to consolidate a difficult diagnosis, as cognitive disorders?
- Do you use screening scores? As the Fagerström?
- Do you use severity score? Like the CRB65?

1. **What are the barriers to using the scores you quoted?**

Items to discuss and associated revival:

- Time
- Knowledge: You know a lot of score?
- Known impact: scores influence your decision-making?
- Relationship: What can think the patient about achieving clinical score in consultation?

1. **what do you think of the future of clinical scores in general practice?**

- With the emergence of the EBM, what is the future of the scores?
- With the multiplicity of tests, how to find your way?
- With the judicialization of medicine, could you be blamed for not having used a score?

1. **how could we improve the scores?**

1. In terms of feasibility?

Shorter, adapted to the general practice, paid...

2. In terms of integration in our activity?

3. Dedicated time?

4. Specific compensation?

5. Delegate the realization (speech therapist for dementia…)?

6. Integrate into medical software?

7. Another?

1. **what would it be "a perfect score?**
